# Gluten in pharmaceutical products: a scoping review

**DOI:** 10.1186/s13643-021-01772-9

**Published:** 2021-08-07

**Authors:** Irene Lizano-Díez, Eduardo L. Mariño, Pilar Modamio

**Affiliations:** Clinical Pharmacy and Pharmaceutical Care Unit, Department of Pharmacy and Pharmaceutical Technology, and Physical Chemistry, Faculty of Pharmacy and Food Sciences, University of Barcelona, Av. Joan XXIII 27-31, 08028 Barcelona, Spain

**Keywords:** Celiac disease, Diet, gluten-free, Excipients, Gluten, Pharmaceutical preparations, Scoping review

## Abstract

**Background:**

Celiac disease (CD) is one of the most common gluten-related disorders. Although the only effective treatment is a strict gluten-free diet, doubts remain as to whether healthcare professionals take this restriction into consideration when prescribing and dispensing medicines to susceptible patients. This scoping review aimed to find out the current evidence for initiatives that either describe the gluten content of medicines or intend to raise awareness about the risk of prescribing and dispensing gluten-containing medicines in patients with CD and other gluten-related disorders.

**Methods:**

A scoping review was conducted using three search strategies in PubMed/MEDLINE, TripDatabase and Web of Science in April 2021, following the PRISMA extension for scoping reviews (PRISMA-ScR). References from included articles were also examined. Two researchers screened the articles and results were classified according to their main characteristics and outcomes, which were grouped according to the PCC (Population, Concept and Context) framework. The initiatives described were classified into three targeted processes related to gluten-containing medicines: prescription, dispensation and both prescription and dispensation.

**Results:**

We identified a total of 3146 records. After the elimination of duplicates, 3062 articles remained and ultimately 13 full texts were included in the narrative synthesis. Most studies were conducted in the US, followed by Canada and Australia, which each published one article. Most strategies were focused on increasing health professional’s knowledge of gluten-containing/gluten-free medications (*n* = 8), which were basically based on database development from manufacturer data. A wide variability between countries on provided information and labelling of gluten-containing medicines was found.

**Conclusion:**

Initiatives regarding the presence of gluten in medicines, including, among others, support for prescribers, the definition of the role of pharmacists, and patients’ adherence problems due to incomplete labelling of the medicines, have been continuously developed and adapted to the different needs of patients. However, information is still scarce, and some aspects have not yet been considered, such as effectiveness for the practical use of solutions to support healthcare professionals.

**Supplementary Information:**

The online version contains supplementary material available at 10.1186/s13643-021-01772-9.

## Background

Celiac disease (CD) is a chronic immune-mediated enteropathy of the small intestine precipitated by exposure to dietary gluten in genetically predisposed individuals [1]. It is one of the most common lifelong disorders worldwide, with a mean prevalence of 1.4% when individuals diagnosed by serologic test are included and an overall prevalence of 0.7% based on biopsy results [2]. Despite the advances in CD diagnosis, in developed countries, for every case diagnosed, an average of five to ten cases remain undiagnosed, usually due to the presentation of atypical, minimal, or even an absence of symptoms. Undiagnosed cases remain untreated, so they are exposed to the risk of presenting other types of complications associated with CD in the long term [3].

Gluten refers to a broad group of prolamins found in wheat, rye and barley [1]. Immune activation of the small bowel due to intolerance to the peptide antigen derived from prolamins produces villous atrophy, crypt hyperplasia and increased intra-epithelial lymphocytes of the lamina propria. At the local level, these changes generate gastrointestinal symptoms and malabsorption [4].

In addition to CD, there are other types of gluten-related disorders, the most important of which is non-celiac gluten sensitivity (NCGS), with intestinal and extra-intestinal symptoms similar to CD [5]. Complete avoidance of gluten intake as well as maintaining a strict and lifetime gluten-free diet (GFD) is the key to reducing symptoms and improving the quality of life of these patients [5].

CD also represents a significant economic burden in terms of healthcare-related expenditures and productivity loss. According to a recent systematic review, healthcare resource utilisation by celiac patients is higher than that of patients without CD, usually because much of the cost of the management derives from outpatient care. These costs drop after diagnosis and adherence to a GFD [6].

Although sensitive patients tend to strictly adhere to a GFD, there is a possibility of unintentional intake via medicines since these may contain gluten in the formulation itself (excipients) or as a result of the manufacturing process (traces) [7]. This amount, which should not be ignored by physicians, pharmacists and patients, could reactivate the small bowel immune response of a sensitive patient.

While patients with gluten intolerance are usually aware of their condition and intake restrictions, there is still doubt about whether healthcare professionals involved in patient treatment share this awareness and are sufficiently informed about the presence of gluten in medicines. Hence, we hypothesise that if current information regarding the awareness and dissemination in clinical practice about the gluten content of medicines is scarce, it must be studied, promoted and developed to ensure patient safety. This scoping review aimed to find out the current evidence for initiatives that either describe the gluten content of medicines or intend to raise awareness about the risk of prescribing and dispensing gluten-containing medicines in patients with CD and other gluten-related disorders. Specifically:

- To identify and describe strategies to increase health professional’s knowledge of gluten-containing/gluten-free medications (e.g. guidelines, database development)
- To assess the knowledge of healthcare professionals (i.e. pharmacists, general practitioners, nurses) regarding CD and the prescription and dispensation of gluten-containing medications (e.g. surveys)
- To identify and describe the barriers to health professionals’ knowledge of gluten-containing/gluten-free medications
- To identify studies that have analysed medicines to determine gluten content
- To assess the knowledge of patients with CD about gluten content of medicines

## Methods

A scoping review was conducted following the “Preferred Reporting Items for Systematic Reviews and Meta-Analyses (PRISMA) extension for scoping reviews (PRISMA-ScR) checklist” (Additional file 1) [8].

### Eligibility criteria

In our literature search, our primary focus was to find studies whose interventions were aimed at identifying gluten-containing medicines, gluten-free manufacturers and also those initiatives that evaluated the degree of awareness among healthcare professionals about gluten-related disorders and the potential harm that gluten-containing medicines may cause in susceptible patients. The ultimate goal of these studies was to facilitate the prescription and/or dispensation of medicines in the gluten-sensitive population. We kept our search broad and utilised the eligibility criteria to narrow down search results in accordance with our predetermined PCC (Population, Concept and Context) framework, which is listed in Table 1 [9].

**Table 1:** PCC framework

Criteria	Description
Population	Human participants Any age Any sex
Concept	Patients with CD and other gluten-related disorders Intervention or informative initiatives aimed at increasing awareness of gluten content of medicines and its potential harm in health outcomes of susceptible patients Intervention or informative initiatives addressed to healthcare professionals and/or patients
Context	Worldwide. No limits on ethnicity or gender All settings considered: outpatients (non-hospitalised, e.g. primary healthcare) and inpatients (hospitalised, acute and subacute) Studies conducted and/or addressed to any healthcare professional involved in diagnosis and management of gluten-related disorders, prescription and/or dispensation of medicines Original research articles (any methods) and review articles, including systematic reviews, meta-analyses, meta-syntheses, narrative reviews, mixed-methods reviews, qualitative reviews and rapid reviews

*CD* celiac disease, *PCC* Population, Concept and Context

Studies had to fulfil the following criteria to be included:

- Studies that identified gluten-containing medicines or gluten-free manufacturers or described initiatives aimed at raising awareness about that condition (i.e. creation of databases)
- Only human medicines
- Protocols/guides/surveys regarding the prescription and/or dispensation of medicines to patients with gluten-related disorders in regular clinical practice
- All searches were conducted without temporal, language or geographical restrictions, since this was a novel review

Exclusion criteria were defined as follows:

- Studies focused on describing comorbidities, diagnosis and/or treatment of CD and other gluten-related disorders, GFD research, gluten-related disorders research, non-clinical research (e.g. basic science, in vitro and/or animal-based research), nutritional/dietary recommendations, research on other food intolerances and/or other allergens
- Any study or initiative that was not related to the prescription or dispensation of medicines which could contain gluten
- Any publication on the gluten content of pharmaceuticals other than medicines (i.e. cosmetics/oral hygiene products, dietary supplements)
- Any publication on the gluten content of food
- Conference abstracts, theses, expert opinions, letters to the editor, websites, books and digital medical applications

### Search strategy

We searched the PubMed/MEDLINE, TripDatabase and Web of Science databases. Three search strategies were designed to identify published studies, using a specific algorithm in accordance with the requirements and characteristics of each database. All search strategies included combinations of the following terms: “celiac disease” [MeSH], “pharmacy research” [MeSH], “pharmaceutical services” [MeSH], “drug utilization review” [MeSH], “glutens” [MeSH], “diet, gluten-free” [MeSH], “medication therapy management” [Mesh], “potentially inappropriate medication list” [MeSH], “Pharmacists” [MeSH], “excipients” [MeSH], “prescription drugs” [MeSH], “nonprescription drugs” [MeSH], “databases, pharmaceutical” [MeSH], “gluten”, “drug utilization review”, “medicines”, “excipients”, “celiac disease”, “inappropriate medication”, “contraindicated medication”, “gluten content of medications”, “prescription or nonprescription”, “pharmaceutical database”.

Each search strategy used for the PubMed/MEDLINE database was identified by Medical Subject Headings (MeSH) terms. However, in order to complete the search and expand the results, additional searches were developed combining free terms (Additional file 2). These search strategies were conducted in April 2021. The same algorithm with the same search strategies was used to extract data from the Web of Science database in April 2021 (Additional file 3). The TripDatabase search strategy was also conducted in April 2021, using the keyword “Gluten” and results were extracted from the “All secondary evidence” category.

In addition, the references included in the studies selected for review were also examined. This complementary search was based on the related articles’ titles and was conducted in Google Scholar.

Two reviewers independently screened all titles and abstracts based on the eligibility criteria defined by the PCC framework. If a title and abstract met the inclusion criteria, then the full text was analysed following the same procedure. Variations in the reviewers’ opinions were resolved through discussion and consensus or consultation with a third reviewer.

### Data extraction

The information extracted from the included studies was the first author’s name, year of publication, country of origin, population according to its underlying disease (i.e. CD, Dermatitis herpetiformis, NCGS), targeted initiative (i.e. prescription, dispensation, both), main outcomes of the study according to the PCC framework (Table 1), number of participants, type of participants (i.e. manufacturers, patients, pharmacists, not applicable) and study design (i.e. descriptive study, non-randomised experimental study, discussion paper, survey). The authors conducted data extraction and analysis using a pre-designed form created using Microsoft Excel (Microsoft Corp).

### Data presentation

The present scoping review followed a narrative approach. Results were synthesised into a narrative summary and tabulated information. Quantitative data of included studies were summarised as numerical counts. Suggestions for future research based on the scoping review findings were also summarised.

Furthermore, a literature map was also designed to illustrate the study design, population, type of participants and targeted process related to gluten-containing medicines.

## Results

**Fig. 1:**
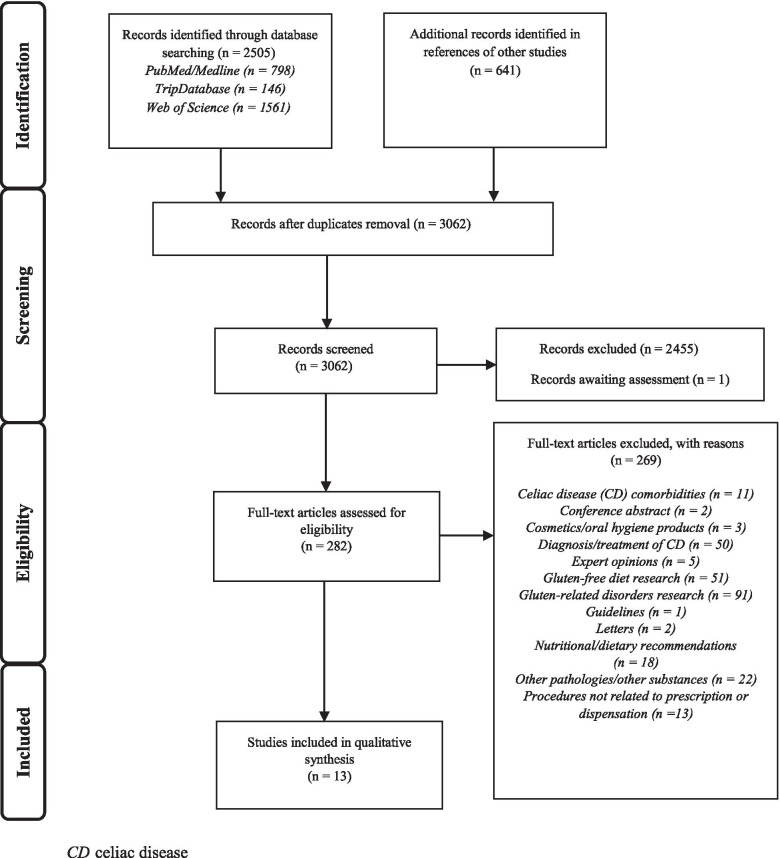
Flow diagram of study selection

We identified a total of 3146 records, 2505 through database search strategies (PubMed/MEDLINE *n* = 798; TripDatabase *n* = 146; Web of Science *n* = 1561) and 641 through references in other studies. After the elimination of duplicates, 3062 articles remained and then 2456 were excluded after the title and abstract screening process. Eligibility assessment was carried out on 282 full texts. At this stage, 269 articles were excluded (reasons are given in Fig. 1), and ultimately 13 full texts were included in the narrative synthesis.

This review included full-text articles published from 1985 to 2021, mostly targeted to CD patients (*n* = 11). They were all written in English. While the first studies about the presence of gluten in medications were published in the 1980s and were from Canada and Australia, the subsequent studies, conducted up through 2021, were all from the United States (US) (*n* = 11).

In total, 5623 patients with gluten-related disorders and 418 pharmacists were surveyed in the included studies. A total of 1239 medications and 700 manufacturers of pharmaceutical products were analysed.

**Fig. 2:**
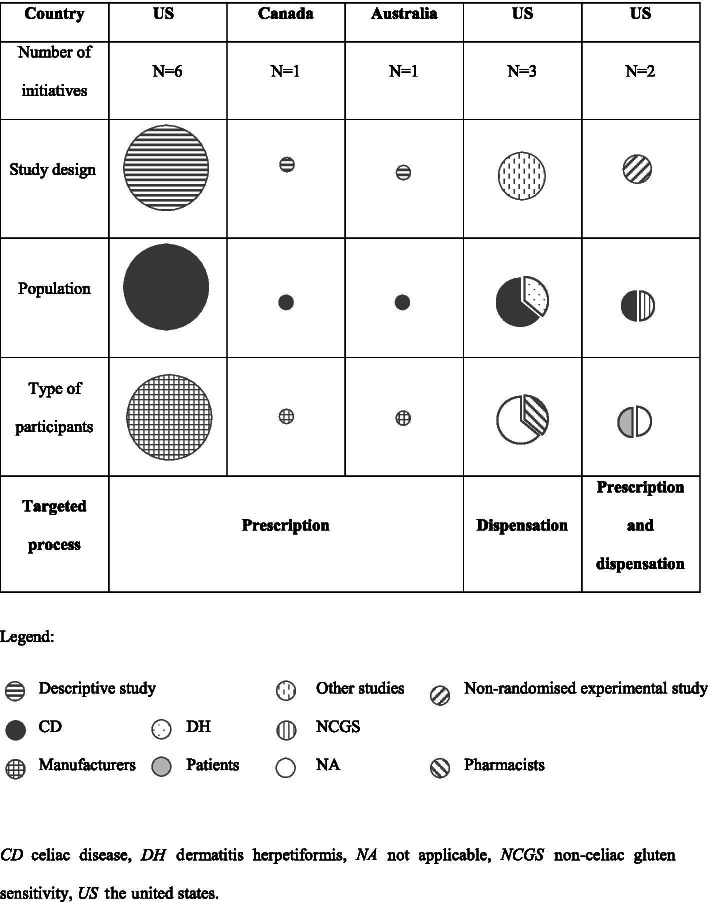
Evidence map of identified literature

Table 2 shows an overview of the characteristics of the included studies, and Fig. 2 describes the mapping of literature.

**Table 2:** Overview of the characteristics of included studies

First author (year)	Country	Population	Targeted process	Main outcomes	No. of participants surveyed	Type of participants	No. of medicines analysed	Study design
Patel (1985) [10]	Canada	CD	Prescription	Development of a gluten-containing medicines database and manufacturers of gluten-free products	73	Manufacturers	103	Descriptive study
Challen (1987) [11]	Australia	CD	Prescription	Development of a gluten-containing medicines database and manufacturers of gluten-free products	68	Manufacturers	214	Descriptive study
Miletic (1994) [20]	US	CD	Prescription and dispensation	Development of a dot blot assay for identification of gliadin presence in medicines	0	NA	59	Non-randomised experimental study
Crowe (2001) [17]	US	CD	Prescription	Development of a gluten-containing medicines database and manufacturers of gluten-free products	105	Manufacturers	340	Descriptive study
Mangione (2008) [7]	US	CD/DH	Dispensation	Review of the epidemiology, pathophysiology, diagnosis, treatment, and complications of celiac disease, in order to provide guidance to pharmacists	0	NA	0	Discussion paper
King (2009) [12]	US	CD	Prescription	Development of a database of manufacturers of gluten-free products	122	Manufacturers	0	Descriptive study
King (2009) [13]	US	CD	Prescription	Follow-up of a previous database of manufacturers of gluten-free products	75	Manufacturers	200	Descriptive study
King (2010) [16]	US	CD	Prescription	Follow-up of a previous database of manufacturers of gluten-free products	121	Manufacturers	0	Descriptive study
Mangione (2011) [18]	US	CD	Dispensation	Review of the aetiology, clinical manifestations, diagnosis, management and presence of gluten in medicines, in order to provide guidance to pharmacists	0	NA	0	Discussion paper
King (2013) [14]	US	CD	Prescription	Development of a gluten-containing medicines database	91	Manufacturers	200	Descriptive study
Jay (2014) [21]	US	CD/NCGS	Prescription and dispensation	Analysis of medicines suspected of causing adverse effects to determine gluten content	5623	Patients	39	Non-randomised experimental study / Survey
Cruz (2015) [15]	US	CD	Prescription	Development of a gluten-containing medicines database	45	Manufacturers	84	Descriptive study
Avena-Woods (2018) [19]	US	CD	Dispensation	Evaluation of the extent of community pharmacists’ self-assessment and actual knowledge of CD	418	Pharmacists	0	Survey

*CD* celiac disease, *DH* dermatitis herpetiformis, *NA* not applicable, *NCGS* non-celiac gluten sensitivity, *US* The United States

### Strategies to increase health professional’s knowledge of gluten-containing/gluten-free medications (n = 8) [10–17]

The first authors who developed an original database in 1985 [10], contacted all Canadian pharmaceutical manufacturers by mail to ask whether their products contained gluten and if they stated that they never used wheat or wheat products as excipients. The researchers then proceeded to create a database of medicines containing gluten and gluten-free manufacturers to support physician decisions about prescribing medicines to CD patients and suggesting that manufacturers should use other excipients and comprehensively label their products.

The same methodology was followed a few years later in Australia (1987) [11], where the major pharmaceutical companies were requested to provide information on the gluten content of their medicines. The researchers advised that it would be necessary to update the information periodically by contacting the manufacturer, as some excipients could change in the future. In addition, the authors pointed out the need to develop a specific regulation for excipients, similar to what existed for food additives in this regard, since their findings revealed that many pharmaceutical products contained wheat starch and therefore residual gluten that could go unnoticed.

A drug information centre in the US developed a list of gluten-free manufacturers in February 2009 [12] and stated that the list would be a good starting place for practitioners when searching for gluten-free medications, and even provided links to some websites with information about the gluten content of medicines. However, since excipients and manufacturing processes can change over time, their advice was that it is essential to check on demand with the manufacturer regarding each specific product. The list was updated by the original authors in November 2009 [13] and July 2012 [14], including the gluten content of the top 200 medications in the US, sorted by retail sales and total number of prescriptions. The authors also included an updated list of manufacturers that claimed to be gluten-free in March 2010 [16].

Likewise, Cruz et al. [15] analysed the manufacturer’s package insert to see if there was a statement indicating the presence of gluten, and if the information was not clearly identified, the manufacturer was contacted. The responses were collected in a database, adding the source and contact data. 70.2% of the responses resulted in inconclusive findings.

In terms of manufacturing policies of gluten-free medications, a survey was carried out in the US (1998) [17]. Only 5 out of 100 pharmaceutical companies reported having a policy for producing gluten-free products. The researchers classified companies into those that indicated if their products were gluten-free and those that indicated they did not add ingredients derived from wheat, oats, barley, rye or spelt to products. The results showed that most of the manufacturers believed that, regardless of not having defined a clear policy to guarantee it, their products were gluten-free, even if they could not guarantee that ingredients purchased from other sources were free from minute amounts of gluten contaminants.

### Knowledge of healthcare professionals regarding CD and the prescription and dispensation of gluten-containing medications (*n* = 3) [7, 18, 19]

Pharmacists’ contribution to medication safety is historically focused on dispensation. Pharmacists have played a key role in determining whether using medications could compromise gluten-free adherence in susceptible patients [7]. Also, it has been described that pharmacists who perceive the signs and symptoms of CD can refer the patient to a physician, thus reducing the time to diagnosis [7, 18]. In order to help pharmacists achieve this goal, Mangione et al. [7] reviewed a broad range of information on the management of CD and also provided advice on how to recognise the gluten content of a medicine. This review was updated in 2011 [18], encouraging healthcare professionals to contact manufacturers to obtain, corroborate or update information on the gluten content of their products.

In order to evaluate the self-assessment and actual knowledge of CD and to identify areas where additional training may be needed, a survey of pharmacists was conducted in 2018 [19]. Results indicated that 95% of all respondents agreed that pharmacists play a role in identifying the disease. However, while many community pharmacists knew the most common facts about CD, 41% of them perceived themselves as having poor or limited knowledge of the disease, and only the 27% that reported their understanding of CD to be basic or advanced correctly defined the disease.

### Barriers to health professionals’ knowledge of gluten-containing/gluten-free medications (*n* = 0)

No studies were retrieved under this category.

### Analysis of medicines to determine gluten content (*n* = 1) [20]

Unlabeled gluten is a concern for patients with CD and related disorders, as a risk of adverse events related to medicines. As a strategy to support healthcare professionals in raising awareness of the necessity for periodic monitoring and assessment of major sources of risk, fifty-nine prescription and non-prescription medicines were analysed in the US in 1994 [20], with the aim of developing a test to detect gliadin in medicines. The assay revealed that gliadins were found in most of the pharmaceutical products tested (71.2%).

### Knowledge of patients with CD about gluten content of medicines (*n* = 1) [21]

In order to characterise the problem of unlabeled gluten and to determine the gluten content of medicines reported to have caused adverse reactions, a survey was conducted of CD and NCGS patients and published in 2014 [21]. Two hundred forty-two different medicines suspected of causing a gluten-related reaction were identified and 39 of them (24 prescription and 15 non-prescription medicines), selected according to the number and severity of reported reactions, how often the medicine was used and the ability to exclude common (non-gluten related) side effects, were tested in duplicate for the presence of gluten. It was found that some medicines (*n* = 3) were over the level of quantification of gluten for the first testing, but there were no medicines over the level of quantification for the second assay.

## Discussion

Most of the initiatives associated with gluten in medicines were targeted to the prescription [10–17], based on practical and user-friendly tools for physicians, such as databases with the gluten content of medicines from manufacturers that could guarantee manufacturing policies of gluten-free products. In the case of dispensation of medicines [7, 18, 19], concerns regarding pharmacists’ knowledge of CD were observed. Consequently, initiatives were aimed at raising awareness and identifying topics that needed further training to guide and support patients. Initiatives that did not make a particular distinction between prescription and dispensation of medicines [20, 21] analysed the presence of unlabeled gluten in medicines through quantitative testing, in order to prevent potential adverse events. It should be noted that studies reporting initiatives addressed to or carried out by other healthcare professionals different from general practitioners or pharmacists were not identified.

Databases with gluten content are intended to help prescribers make informed decisions. However, it is recommended that they be continuously updated by directly contacting the manufacturer, since raw materials or some formulation procedures could be different from those used when the data was originally collected [11–14, 16] and because, although many manufacturers believed that their products were devoid of gluten, they did not certify or test the gluten-free status in the final products [15, 17].

Pharmacists must support patients with CD and other gluten-related disorders, and they should be prepared to inform them about GFD and resolve all kinds of concerns about the disease [7, 18]. A targeted case finding service for CD showed that patients commented on the professionalism exhibited by the pharmacists and on the usefulness of the information provided in a service for CD [22], adding value to the relationship between patients and their local pharmacists and confirming that the upskilling of the healthcare professional can be a valuable tool [19, 23].

A lack of clarity in the labelling of medicines could be a risk for susceptible patients, possibly leading to an adherence problem, as a patient may not adhere to a prescribed regimen or the patient’s therapy may be changed because of unknown or suspected information, even if the medicine showed no presence of gluten after a quantitative analysis [20, 21]. As shown by other authors, some patients experienced anxiety and they did not adhere to prescribed medication regimens, ending a treatment against medical advice due to suspicion of unintentional gluten intake from excipients [24].

There is wide variability between countries on provided information and labelling of gluten-containing medicines. Definitely, the most cost-effective and easiest way to provide information on whether a medicine contains gluten or not would be including a clear statement in the packaging (i.e. quantity, source). In this regard, the European Medicines Agency (EMA) and other national agencies such as The Spanish Agency of Medicines and Medical Devices (AEMPS) have been developing and updating medicine labels, as well as publishing gluten status databases and public access guidelines with information for health professionals and patients [25, 26]. The declaration of gluten content is mandatory for those member states and drug manufacturers which are under the umbrella of the EMA. For instance, the statement “Gluten-free” applies only if the gluten content in the medicinal product is less than 20 parts per million (ppm). However, the US Food and Drug Administration (FDA) only recommends that drug manufacturers use the following statement about gluten, “Contains no ingredient made from a gluten-containing grain (wheat, barley, or rye)”, when it is truthful and substantiated, according to a draft guidance that contains nonbinding recommendations [27].

Table 3 summarises the findings identified in the scoping review, as well as the future challenges of the different stakeholders involved in the management of patients with gluten-related disorders, from healthcare professionals to patients, including manufacturers and medicines agencies. Therefore, it is clear that different initiatives to raise awareness among healthcare professionals have been developed, although the usefulness of these initiatives has not yet been evaluated. There is room for improvement and further studies should be developed with more robust study designs, such as prospective observational studies, since it is necessary to confirm the effectiveness of the initiatives and the impact on the outcomes and quality of life of susceptible patients.

**Table 3:** Current findings and future challenges

Findings focused on patients with gluten-related disorders	Future challenges
**Healthcare professionals**	
• Need to update gluten content databases to support prescription [10–17] • Definition of the role of pharmacists in the management of susceptible patients [7, 18] • Limited knowledge of pharmacists about celiac disease and its management [19] • Studies reporting initiatives addressed to or carried out by other healthcare professionals different from general practitioners or pharmacists were not identified	• Computerised decision-making support systems resulting in the generation of patient-specific assessments and recommendations for clinicians (e.g. algorithms and integrated alerts in the electronic prescription system) • Continuous disease-specific training and professionalisation of pharmaceutical care services (i.e. review of content for prescribed medicines in susceptible patients, and also non-prescription medicines and other pharmaceutical products) • Interoperability of electronic medical records between primary healthcare and pharmacies in order to identify susceptible patients before dispensation of medicines or other pharmaceuticals that could potentially contain gluten • Further robust studies are needed to assess the effectiveness of initiatives and strategies to increase awareness of gluten content of medicines
**Patients**	
• Mistrust in manufacturers and/or healthcare professionals in case of adverse events related to gluten content of the medicines prescribed [21] • Unnoticed gluten intake through medicines [21]	• Patient training and empowerment to facilitate self-management, knowledge about the disease and the potential safety risks • Promoting a closer relationship with healthcare professionals and a collaborative partnership to ensure patients’ needs and preferences • Further robust studies are needed to assess the effectiveness of initiatives and strategies to increase awareness of gluten content of medicines as well as patient reported outcomes (i.e. quality of life, satisfaction)
**Manufacturers**	
• Need to update the lists of manufacturers that never use gluten as excipient of medicines [10, 16, 17] • Awareness on incomplete medicine labels that could lead to adherence problems for patients and other safety issues (e.g. adverse events) [10–15, 17, 20] • Lack of gluten-free manufacturing policies [17] • Variability among countries on provided information and labelling of gluten content of medicines [10–17]	• Detailed list of all medicine components, explicit labelling in case of potentially harmful ingredients • Development of inherently gluten-free medicines and trace testing in final product • Inclusion of a wider spectrum of patients in clinical trials and design of development programmes for patients with celiac disease and other gluten-related disorders • Fostering worldwide cooperation and harmonisation (e.g. development of generic policies in relation to information on gluten content of medicines)
**Medicines agencies**	
• Responsibility for gluten-free medicines policies [25, 27]	• Specific guidelines help to ensure a safe use of medicines available to patients with celiac disease and other gluten-related disorders and also to healthcare professionals • Fostering worldwide cooperation and harmonisation (e.g. development of generic policies in relation to information on gluten content of medicines) • Strategies for funding and maintaining a gluten-containing medicines research

This scoping review is subject to certain limitations since there are very few publications on this subject, many of which were written more than 30 years ago. The true extent of the initiatives could be underestimated, since grey literature was not explored. In addition, the results incorporated in this scoping review derive from a heterogeneous set of studies with different methodologies used. In this regard, the methodology of the analysis was not clearly described in some publications, or the results were presented as aggregated, and thus could not be analysed in detail. Furthermore, some studies could not be directly compared because of the diversity of countries and periods analysed.

## Conclusions

Initiatives that raise awareness of prescription and dispensation of gluten-containing medicines have been designed for more than 35 years and most of the efforts have focused on the prescription process, through practical tools that should be continuously updated. However, in the last decades, the dispensation process has been considered as critical as prescribing and research was aimed at improving pharmacists’ knowledge of CD, highlighting the problem of unintentional gluten intake from medicines and their potential adverse effects.

These initiatives establish that collaborative objectives are necessary to address safe therapy for patients with gluten-related disorders and are the first step towards the development of further studies whose objectives should be focused on updating initiatives that take into account the gluten content of medicines, as well as measuring the effectiveness in daily healthcare professionals’ practice.

Future studies such as randomised controlled trials and observational studies are needed, including specific interventions related to inadvertent prescription and/or dispensation and/or use of gluten-containing medicines that will help by adding more evidence and raising awareness about adverse drug events at the level of healthcare professionals as well as health consumers. The inclusion of other relevant patient-reported outcomes like quality of life and satisfaction would be also recommended.

In addition, it is important to establish a collaborative model between community pharmacy and primary healthcare to complete the patient’s journey. For instance, integrated alerts in the electronic prescription system could be established to support the prescription and dispensation processes, and thus avoid the consumption of medications that may contain gluten by susceptible patients.

## Supplementary Information

**Additional file 1.** PRISMA-ScR Checklist.

**Additional file 2.** PubMed/MEDLINE database search strategy.

**Additional file 3.** Web of Science database search strategy.

## Data Availability

The search strategies for PubMed/MEDLINE and Web of Science are described in the Appendices. Other datasets used and/or analysed during the scoping review are available from the corresponding author on reasonable request.

## Abbreviations

AEMPS: The Spanish Agency of Medicines and Medical Devices
CD: Celiac disease
DH: Dermatitis herpetiformis
EMA: European Medicines Agency
FDA: United States Food and Drug Administration
GFD: Gluten-free diet
MeSH: Medical Subject Headings
NA: Not applicable
NCGS: Non-celiac gluten sensitivity
PCC: Population, Concept and Context
Ppm: Parts per million
PRISMA: Preferred Reporting Items for Systematic Reviews and Meta-Analyses
PRISMA-ScR: PRISMA extension for scoping reviews
US: The United States
