# Supplementary material for: Gluten in pharmaceutical products: a scoping review
Source: Syst Rev. 2021 Aug 7;10:218. doi: 10.1186/s13643-021-01772-9 (PMC8349483; doi:10.1186/s13643-021-01772-9)
Supplement: Supplementary file 2 — Additional file 2. PubMed/MEDLINE database search strategy. [file 13643_2021_1772_MOESM2_ESM.docx]

**Additional file 2**. PubMed/Medline database search strategy.

| **Search** | **Search query** |
| --- | --- |
| #1 | ("Celiac Disease"[MeSH]) AND (("Pharmacy Research"[MeSH] OR "Pharmaceutical Services"[MeSH])) |
| #2 | ("Celiac Disease"[MeSH]) AND "Drug Utilization Review"[MeSH] |
| #3 | (((("Glutens"[MeSH]) OR "Diet, Gluten-Free"[MeSH]) OR "Celiac Disease"[MeSH])) AND "Drug Utilization Review"[MeSH] |
| #4 | (GLUTEN) AND Drug Utilization Review |
| #5 | GLUTEN AND medicines |
| #6 | GLUTEN AND excipients |
| #7 | (((("Glutens"[MeSH]) OR "Diet, Gluten-Free"[MeSH]) OR "Celiac Disease"[MeSH])) AND (("Medication Therapy Management"[MeSH]) OR "Potentially Inappropriate Medication List"[MeSH]) |
| #8 | (Celiac Disease OR gluten) AND (Inappropriate Medication) |
| #9 | (Celiac Disease OR gluten) AND (CONTRAINDICATED Medication) |
| #10 | (((("Glutens"[MeSH]) OR "Diet, Gluten-Free"[MeSH]) OR "Celiac Disease"[MeSH])) AND (("Pharmacy Research"[MeSH]) OR "Pharmacists"[MeSH]) |
| #11 | gluten content of medications |
| #12 | ((("Celiac Disease"[MeSH]) AND "Excipients"[MeSH])) AND (((("Prescription Drugs"[MeSH]) OR "Nonprescription Drugs"[MeSH])) AND "Databases, Pharmaceutical"[MeSH]) |
| #13 | "Celiac Disease"[MeSH] AND (("Prescription Drugs"[MeSH] OR "Nonprescription Drugs"[MeSH]) OR "Databases, Pharmaceutical"[MeSH]) |
| #14 | (Celiac Disease OR gluten) AND (prescription or nonprescription) |
| #15 | (Celiac Disease OR gluten) AND (pharmaceutical database) |

Note: Search strategy string was the following (#1 OR #2 OR #3 OR #4 OR #5 OR #6 OR #7 OR #8 OR #9 OR #10 OR #11 OR #12 OR #13 OR #14 OR #15).
