# Supplementary material for: Gluten in pharmaceutical products: a scoping review
Source: Syst Rev. 2021 Aug 7;10:218. doi: 10.1186/s13643-021-01772-9 (PMC8349483; doi:10.1186/s13643-021-01772-9)
Supplement: Supplementary file 3 — Additional file 3. Web of Science database search strategy. [file 13643_2021_1772_MOESM3_ESM.docx]

**Additional file 3**. Web of Science database search strategy.

| **Search** | **Search query** |
| --- | --- |
| #1 | ALL= (Celiac Disease AND (Pharmacy Research OR Pharmaceutical Services)) |
| #2 | ALL= (Celiac Disease AND Drug Utilization Review) |
| #3 | ALL= (((Gluten*) OR (Diet, Gluten-Free) OR (Celiac Disease)) AND (Drug Utilization Review)) |
| #4 | ALL= (GLUTEN* AND Drug Utilization Review) |
| #5 | ALL= (GLUTEN* AND MEDICINE*) |
| #6 | ALL= (GLUTEN* AND EXCIPIENT*) |
| #7 | ALL= (((Gluten*) OR (Diet, Gluten-Free) OR (Celiac Disease)) AND ((Medication Therapy Management) OR (Potentially Inappropriate Medication List))) |
| #8 | ALL= (((Celiac Disease) OR (gluten*)) AND (Inappropriate Medication*)) |
| #9 | ALL= (((Celiac Disease) OR (gluten*)) AND (CONTRAINDICATED Medication*)) |
| #10 | ALL= (((Gluten*) OR (Diet, Gluten-Free) OR (Celiac Disease)) AND ((Pharmacy Research*) OR (Pharmacist*))) |
| #11 | ALL= gluten content of medication* |
| #12 | ALL= (((Celiac Disease) AND (Excipient*) AND (Prescription Drug*)) OR (Nonprescription Drug*) AND (Database*, Pharmaceutical)) |
| #13 | ALL= ((Celiac Disease OR (gluten*)) AND (prescription or nonprescription)) |
| #14 | ALL= ((Celiac Disease OR (gluten*)) AND (pharmaceutical database*)) |

Note: Search strategy string was the following (#1 OR #2 OR #3 OR #4 OR #5 OR #6 OR #7 OR #8 OR #9 OR #10 OR #11 OR #12 OR #13 OR #14).
